# Multifocal cholesterol granulomas of the anterior mediastinum

**DOI:** 10.1186/s40792-020-00943-5

**Published:** 2020-07-28

**Authors:** Takehiko Manabe, Soichi Oka, Kenji Ono

**Affiliations:** Thoracic surgery, Kokura Memorial Hospital, Asano, Kokurakita-ku, Kitakyushu-shi, Fukuoka, 802-8555 Japan

**Keywords:** Cholesterol granuloma, Mediastinal tumor

## Abstract

**Background:**

Cholesterol granuloma in the mediastinum is rarely observed, accounting for 1% of all mediastinum tumors. There have been only a few reports of multifocal cholesterol granulomas of the thymus. We herein report a rare case of multifocal cholesterol granuloma in the thymus that was incidentally detected during follow-up of an aortic aneurysm.

**Case presentation:**

The patient was a 70-year-old man with dyslipidemia and hypertension who was referred to our hospital to undergo an operation for chest aortic aneurysm. Preoperative computed tomography (CT) showed 4 lesions in the anterior mediastinum measuring up to 4 cm in size with slight contrast enhancement and spotty calcification. Therefore, a thymoma, bronchogenic cyst, or lymphangioma were considered as the preoperative diagnosis. The patient underwent total thymectomy under thoracotomy followed by aortic arch replacement for the aortic aneurysm. The pathological diagnosis was multifocal cholesterol granulomas in the thymus.

**Conclusions:**

Cholesterol granulomas should be included in the differential diagnosis of cystic tumor in the mediastinum, especially in patients with basal disease such as dyslipidemia and hypertension, which may lead to aortic aneurysm. Furthermore, complete surgical resection and a detailed histological evaluation are important for the accurate diagnosis and treatment.

## Background

The occurrence of cholesterol granuloma of the mediastinum is extremely rare, but it is common to find cholesterol granulomas in the mastoid antrum and air cells of the temporal bone [1]. We herein report a rare case of a patient who histopathologically presented with cholesterol granuloma formation of the superior and anterior mediastinum.

## Case presentation

**Fig. 1:**
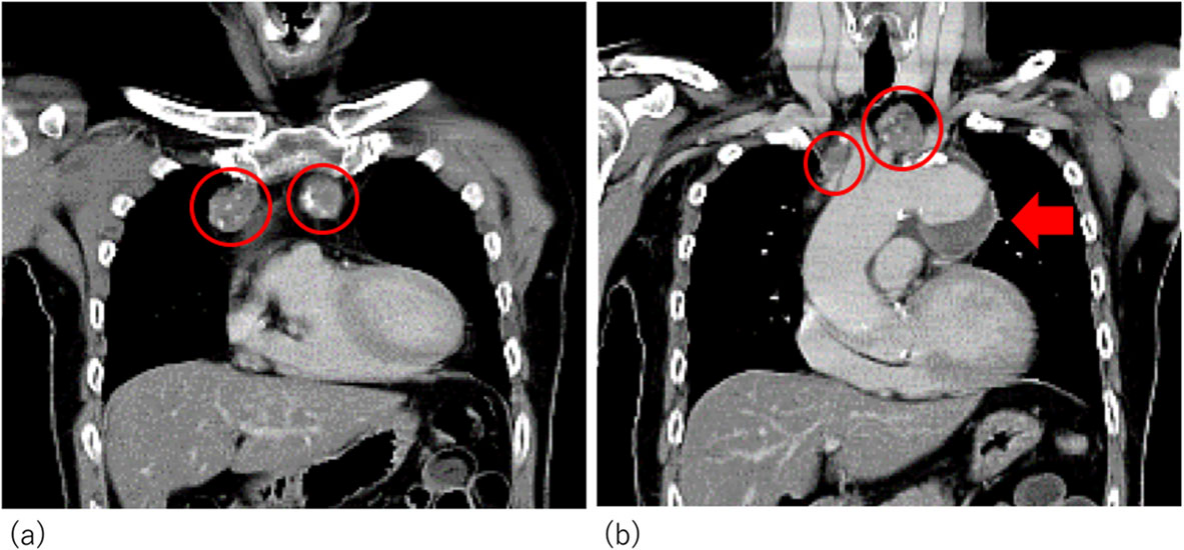
Computed tomography revealed four lesions in the anterior mediastinum (circles) and an aortic aneurysm (arrow)

The patient was a 70-year-old male who was referred to our hospital to undergo an operation for an aneurysm of the aortic arch. He had slight hoarseness that was suspected of being caused by the aortic aneurysm. His medical history included dyslipidemia and hypertension. Preoperative computed tomography showed different-size multifocal lesions in the mediastinum from the anterior to the superior region with slight contrast enhancement, low-density areas equivalent to fat tissue, and spotty calcification (Fig. 1). Laboratory data showed a slightly high level of LDL-cholesterol (216 mg/dL), triglyceride (170 mg/dL), and soluble IL-2R (496 U/ml). Other tumor markers, including carcinoembryonic antigen (CEA), alpha fetoprotein (AFP), and human chorionic gonadotropin (HCG), were within the normal ranges. He had no history of trauma and was not on any anticoagulant drugs. In addition, angiography showed stenosis on the coronary artery, and head magnetic resonance imaging (MRI) showed an asymptomatic old cerebral infarction.

**Fig. 2:**
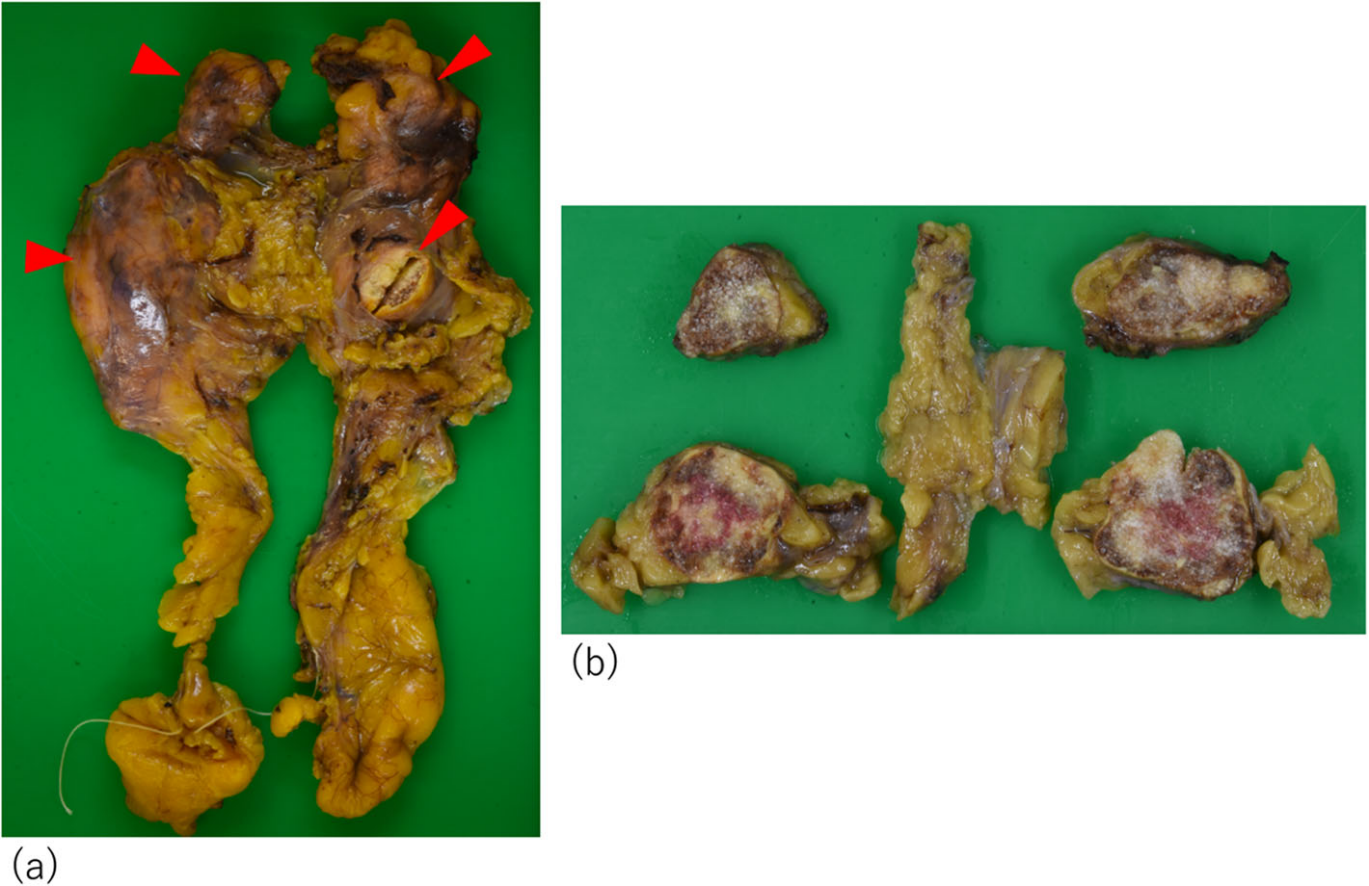
**a** Macroscopically, the nodules were 4.2, 2.3, 2.6, and 3.2 cm in diameter (arrowheads). **b** The cut surface of one of the nodules revealed a yellowish-brown, elastic, hard nodule in the thymus

**Fig. 3:**
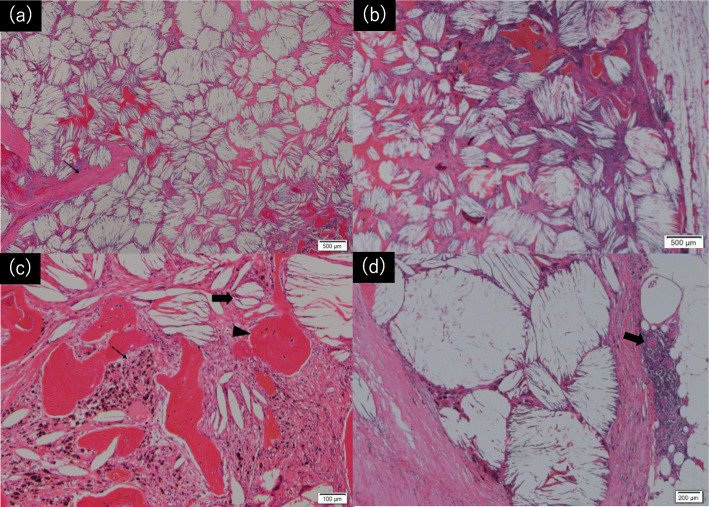
**a** Microscopically, the nodule was identified as a cholesterol granuloma with cholesterol clefts arranged in an alveolar-like growth pattern. Fibrosis was observed in part of the lesion (small arrow). **b** Areas containing inflammatory infiltrates and multinucleated giant cells were observed in the granuloma. **c** The cholesterol clefts (large arrow) were surrounded by inflammatory infiltrates, histocytes, and multinucleated giant cells, some of which had phagocytosed hemosiderin granules (small arrow). In addition, osseous metaplasia (arrowhead) was observed close to the cholesterol clefts. **d** The cholesterol granuloma was adjacent to the remnants of the thymic tissue with the presence of Hassall’s corpuscles (large arrow)

Because we suspected the tumors of being multiple thymoma or malignant lymphoma, we decided to remove the tumor for a definitive diagnosis and treatment. We performed total thymectomy followed by aortic arch replacement and coronary artery bypass under median sternotomy. During the surgery, we observed four lesions in the thymus with elastic hardness. One of the granulomatous masses was located at the caudal of the left lobe of the thyroid gland. The tumors were 4.2 × 2.6 × 1.7 cm, 2.3 × 2.3 × 1.4 cm, 2.6 × 2.6 × 2.4 cm, and 3.2 × 3.0 × 2.2 cm in size with yellowish-brown discoloration, and they were located on the left side, right side, top, and bottom of the thymic tissue (Fig. 2). In addition, a microscopic examination of these tumors revealed a series of cholesterin crystals surrounded by multinucleated giant cells and histocytes, some of which had phagocytosed hemosiderin granules, findings that were consistent with a cholesterol granuloma (Fig. 3a). Osseous metaplasia and calcification were also observed in the tumors (Fig. 3b). The granuloma was adjacent to the remnants of thymic tissue, although there was no particular evidence showing that the thymic tissue had penetrated the granuloma.

Based on these findings, the pathological diagnosis of cholesterol granuloma in the thymus was made. The patient was discharged without any postoperative complications and was followed up for 2 months postoperatively without any recurrence.

## Discussion

Cholesterol granuloma is a benign entity comprising a foreign-body giant cell reaction that forms in response to the presence of cholesterol crystals [1, 2]. It was first described by Manasse et al. in 1894 [3]. It is frequently described as occurring in the temporal bone as a result of inflammatory ear disease. In addition, some clinical studies have reported that these lesions develop in the breast, peritoneum, kidney, testis, mediastinum, liver, spleen, thyroid, and parotid gland [4].

Our search of the literature revealed 12 cases of cholesterol granulomas occurring in the mediastinum, including the present case. The patients were 10 men and 2 women, ranging in age from 25 to 75 years old with a mean age of 61.8 years old [1, 2, 5–12] (Table 1). The clinical presentations were variable, and some of the cases were encountered incidentally, such as the present case. In three cases, the granulomas were detected during cardiac surgery in the absence of symptoms. Regarding the background of the patients, four had medical histories of dyslipidemia. The sites of the tumors were mostly the anterior mediastinum, but one case showed a tumor in the posterior mediastinum, which had been suspected of being a bronchogenesis cyst. Only in two cases, including our own, did the lesions multifocally occur in the mediastinum. In our case, based on the clinical findings, we suspected him of having thymoma, a bronchogenesis cyst, or lymphangioma. Since it was difficult to make a histological diagnosis of the tumor preoperatively, surgical resection should be considered for the accurate diagnosis and treatment.

**Table 1:** Previously reported cases of cholesterol granuloma in the mediastinum

Authors	Age/sex	Background	Site	Size (cm)/mono or multifocal	Radiological findings	Surgical procedure
Luckraz et al. [1]	74/M	Aortic stenosis	Anterior	0.7 × 2.0 × 2.7/mono	No data	Extirpation through median sternotomy
Fujimoto et al. [2]	62/M	Myocardial infarction, thrombosis in the left atrium, dyslipidemia, on warfarin	Anterior	2.0 × 1.8/mono	CT: slight contrast enhancement, spotty calcification within the tumor; PET: an increased uptake; MRI: low signal intensity on both T1- and T2-weighted images	Extirpation through median sternotomy
Shindo et al. [5]	55/M	None	Posterior	4.5 × 7.0/mono	CT: non-contrast enhancement, MRI: high signal intensity on both T1- and T2-weighted images	Extirpation through VATS
Ezzat et al. [6]	75/M	Angina, hypertension, peripheral vascular disease, COPD, dyslipidemia, smoker, trauma	Anterior	2.0 × 3.0 × 3.0/mono	No data	Extirpation through median sternotomy
Krishman et al. [7]	65/M	Third degree heart block, pericarditis, hypertension, coronary artery stenosis	Anterior	1.0 × 1.9 × 2.0/mono	No data	Extirpation through median sternotomy
Ghigna et al. [8]	53/M	None	Anterior	2.0 × 3.0 × 5.0/mono	No data	Thymectomy through median sternotomy
Ghigna et al. [8]	25/M	Car racer	Anterior	1.2 × 2.5 × 4.0/mono	No data	Thymectomy through median sternotomy
Kawai et al. [9]	68/F	Chronic thyroiditis	Anterior	1.0 × 2.0/mono	CT: non-contrast enhancement, scattered nodules within the tumor; PET: an increased uptake; MRI: low signal intensity on both T1- and T2-weighted images	Extended thymectomy through median sternotomy
Drury et al. [10]	74/M	COPD, atrial fibrillation	Anterior	3.2/mono	CT: focal calcification, PET: an increased uptake	Thymectomy through VATS
Kobayashi et al. [11]	64/F	Diabetes mellitus	Anterior	2.0 × 1.7/mono	CT: non-contrast enhancement, MRI: low signal intensity on both T1- and T2-weighted images	Thymectomy through VATS
Nagata et al. [12]	56/F	Dyslipidemia	Anterior	2.0 × 2.0 × 1.0, 1.3 × 1.3 × 1.2/multi	CT: non-contrast enhancement, PET: an increased uptake	Extended thymectomy through median sternotomy
Our case (2020)	71/F	Dyslipidemia, hypertension, aortic aneurysm, coronary artery stenosis, old cerebrovascular infarction	Anterior	4.2 × 2.6, 3.2 × 3.0; 2.6 × 2.6, 2.3 × 2.3/multi	CT: slight contrast enhancement, spotty calcification within the tumor	Total thymectomy through median sternotomy

The preoperative diagnosis of mediastinal cholesterol granuloma may be challenging due to its rarity and radiological appearances resembling common mediastinal tumors such as thymoma. In some reports, the lesions had low-density areas equivalent to fat tissue, scattered nodules, and spotty calcifications on chest CT images [6, 9]. Positron emission tomography shows an increased glucose uptake, probably related to the granulomatous inflammation. The favored radiologic examination is a magnetic resonance imaging, which generally emits a high signal intensity on both T1- and T2-weighted scans. This high signal intensity, which is due to the peripheral accumulation of free methemoglobin, can be amplified by intravenous gadolinium [1].

Several theories have been proposed concerning the pathogenesis of cholesterol granuloma, but the details remain unclear. According to one major theory concerning cholesterol granulomas, local hemorrhaging potentially resulting from trauma or during an inflammatory response causes degeneration of the cells, which develop cholesterol crystals. These cholesterol crystals then induce a foreign-body-type giant cell reaction that is responsible for the granuloma formation [7].

Dyslipidemia is a risk factor of aortic aneurysm [13]. High levels of low-density lipoprotein cholesterol and triglyceride levels lead to atherosclerosis, which causes weakening of the aortic wall and results in the development of aneurysms. Around 10% of patients diagnosed with aortic aneurysm have multiple aneurysms in different segments of the aorta [14]. The pathological findings in our patient showed cholesterol clefts surrounded by inflammatory infiltrates, histocytes, and multinucleated giant cells, some of which had phagocytosed hemosiderin granules. Since these findings indicate microbleeding and chronic inflammation, we can assume that weakness of the vessel walls, as is typical with aneurysms, led to the development of cholesterol granuloma. Furthermore, our case report and search of the literature (Table 1) suggested that cholesterol granulomas were prone to develop in patients with dyslipidemia and hypertension who had a basic condition that could cause vascular disease, such as aortic aneurysm, coronary artery disease, and cerebrovascular disease. Whether or not a relationship exists between cholesterol granuloma and vascular disease is unclear at present, and more research is needed to clarify the relationship between these entities.

However, another theory concerning the pathogenesis has also been proposed. Weissferdt et al. [15] reported four cases of mediastinal tumors dubbed thymic cholesteroloma, which is a kind of cholesterol granuloma of mediastinum. This lesion is characterized by residual thymic tissue at the periphery of the formation of cholesterol cleft granulomas. Weissferdt et al. proposed a different theory concerning its pathogenesis from cholesterol granuloma: the growth of cholesterol cleft granulomas may be induced by the rupture and inflammation of a benign cystic tumor. In their study, they performed complete surgical resection for all of their cases. In our case, we were unable to detect a clear relationship between the thymus and the tumor, although there was residual thymic tissue that existed adjacent to the granulomas. Complete surgical resection and a detailed histological evaluation are important for making an accurate diagnosis, as little is known about the etiology of this tumor.

## Conclusions

We herein report a rare case of multifocal cholesterol granulomas of the anterior mediastinum with aortic aneurysm. For the accurate diagnosis and treatment, cholesterol granulomas should be included in the differential diagnosis of cystic tumors in the mediastinum, especially in patients with a relevant basal disease, such as dyslipidemia and hypertension that can lead to an aneurysm. Although its pathogenesis remains unclear, surgical resection and a detailed histological evaluation are important for the accurate diagnosis and treatment.

## Data Availability

Not applicable

## Abbreviations

CT: Computed tomography
LDL: Low-density lipoprotein
IL-2R: Interleukin-2 receptor
CEA: Including carcinoembryonic antigen
AFP: Alpha fetoprotein
(AFP) HCG: Human chorionic gonadotropin
MRI: Magnetic resonance imaging
